# Bridging Therapy for Acute Stroke as the Initial Manifestation of Takayasu Arteritis: A Case Report and Review of Literature

**DOI:** 10.3389/fimmu.2021.630619

**Published:** 2021-04-30

**Authors:** Yongqiang Dai, Yi Zhong, Banghao Jian, Ping Liu, Kangyu Song, Yu Li, Aimin Wu, Bingjun Zhang

**Affiliations:** ^1^ Department of Neurology, Center for Mental and Neurological Disorders and Diseases, The Third Affiliated Hospital of Sun Yat-sen University, Guangzhou, China; ^2^ Department of Dermatology, Guangzhou Women and Children’s Medical Center, Guangzhou, China

**Keywords:** takayasu arteritis, stroke, bridging therapy, endovascular therapy, emergency

## Abstract

Takayasu arteritis (TA) is a chronic inflammatory disease involving the aorta and its principal branches. Acute ischaemic stroke (AIS) as the initial manifestation of TA is uncommon. There is little evidence on the efficacy of bridging therapy for AIS induced by TA. A 23-year-old Chinese woman with a suspected stroke presented to our hospital with sudden onset of right-sided weakness, right facial palsy, and aphasia that occurred 1 hour ago. After physical and ancillary examinations, recombinant tissue plasminogen activator was administered to the patient, which led to partial recovery. Her neurological function deteriorated with a large salvageable ischaemic penumbra on computed tomography perfusion. Cerebrovascular angiography showed multiple stenoses in the brachiocephalic trunk, the beginning of the right common carotid artery (CCA), and the bilateral subclavian arteries, as well as occlusion of the left CCA and its branches. Mechanical thrombectomy of the left middle cerebral artery was performed immediately. Percutaneous transluminal balloon angioplasty of the left CCA followed by stent implantation of the proximal left CCA was then performed. A diagnosis of TA was made based on the findings. The patient’s neurological deficit fully recovered with immunosuppressants at the 3 month-follow-up. We report a rare case of a patient with TA initially presenting with AIS treated with bridging therapy with full recovery of neurological function. Bridging therapy should be taken into consideration for AIS in patients with TA. Further study is needed in this regard.

## Background

Takayasu arteritis (TA) is a chronic large vessel inflammatory disease involving the aorta and its principal branches, leading to stenosis and occlusion of the vessels (1). Depending on the location and severity of the lesions, its clinical manifestations can vary significantly, from the patient being asymptomatic to occurrence of severe vascular events (2). The incidence of stroke in TA is estimated to be between 10% and 20% (3, 4). However, stroke as the initial manifestation of TA is uncommon (5).

Although mechanical thrombectomy and/or thrombolysis therapy are the gold standard treatments for acute ischaemic stroke (AIS) within the time window, there is little published evidence on mechanical thrombectomy and/or thrombolysis therapy for AIS induced by TA. Hence, we present a case of TA presenting with AIS as an initial manifestation, which was successfully treated with bridging therapy with intravenous thrombolysis and subsequent mechanical thrombectomy.

## Case Presentation

**Figure 1:**
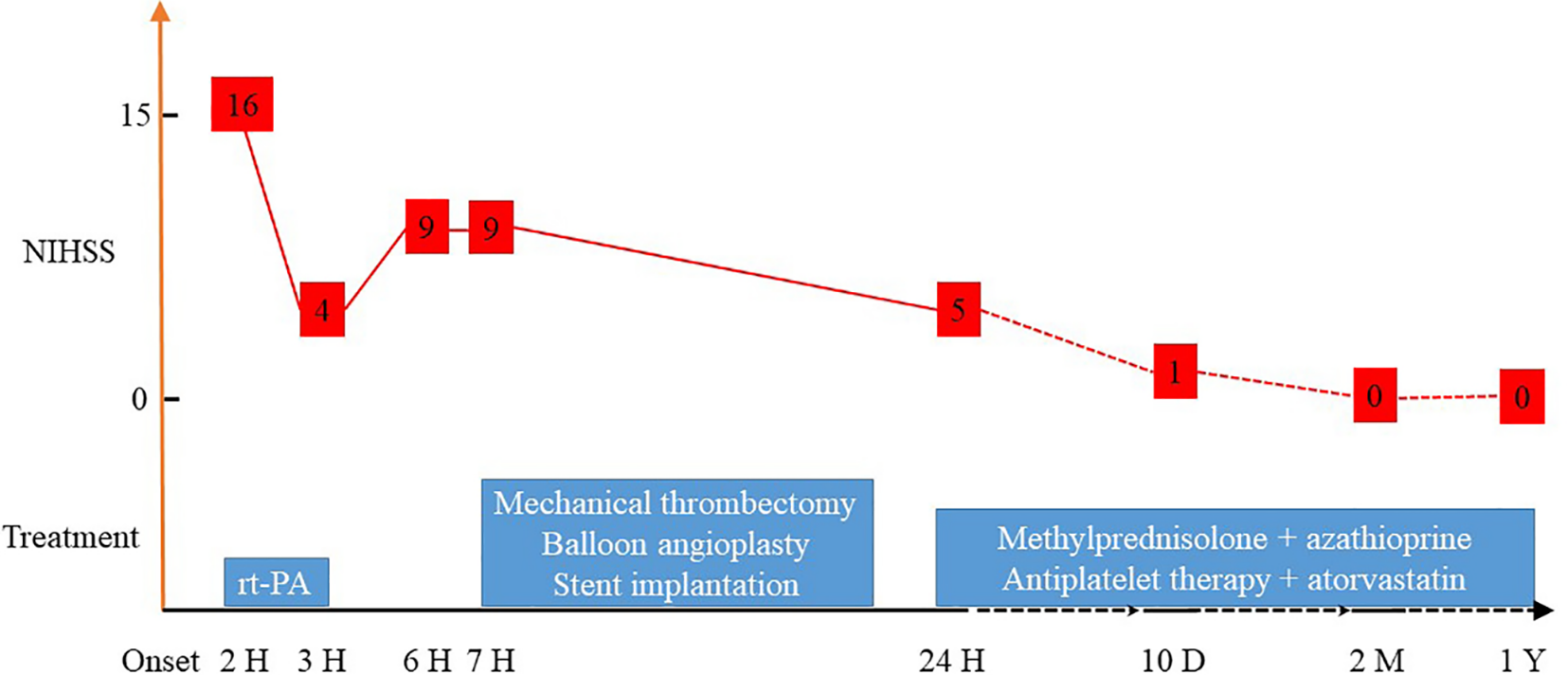
NIHSS scores after different treatments. NIHSS, National Institutes of Health Stroke Scale.

**Figure 2:**
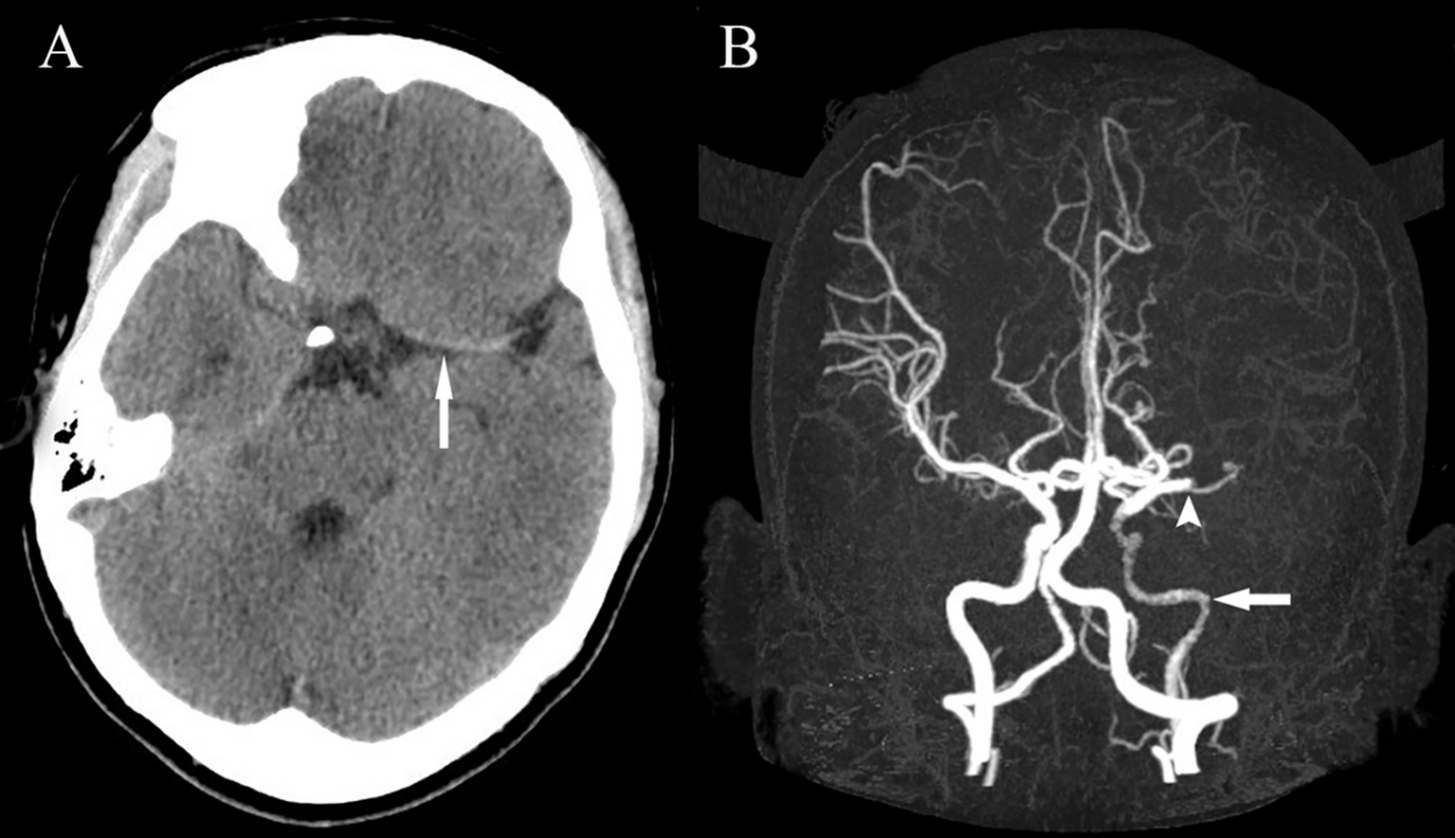
Head CT showing a hyperdense MCA sign involving the M1 segment and the M2 branch of the left MCA **(A)**. Head CTA showing internal carotid artery asymmetry (left smaller than the right) and abrupt occlusion of the M1 branch of the left MCA **(B)**. CT, computed tomography; CTA, computed tomography angiography; MCA, middle cerebral artery.

A 23-year-old Chinese woman with no significant medical history presented to the emergency department with a 1-hour sudden onset of right-sided weakness, right facial palsy, and aphasia. She was not under any medication and had no known family history, including cardiac, cerebrovascular, or other chronic diseases. During admission, a blood pressure difference between the arms was noted, with the left arm at 112/65 mmHg and the right arm at 99/60 mmHg. There was no appreciable carotid bruit, with the electrocardiogram showing regular sinus rhythm. A physical examination revealed absent left carotid pulse, complete aphasia, right-sided central facial palsy, and right-sided hemiplegia (limb power grade 0/5). The initial National Institutes of Health Stroke Scale (NIHSS) score was 16 (**Figure 1**). Head computed tomography (CT) showed a hyperdense middle cerebral artery (MCA) sign involving the M1 segment and the M2 branch of the left MCA (**Figure 2A**).

**Figure 3:**
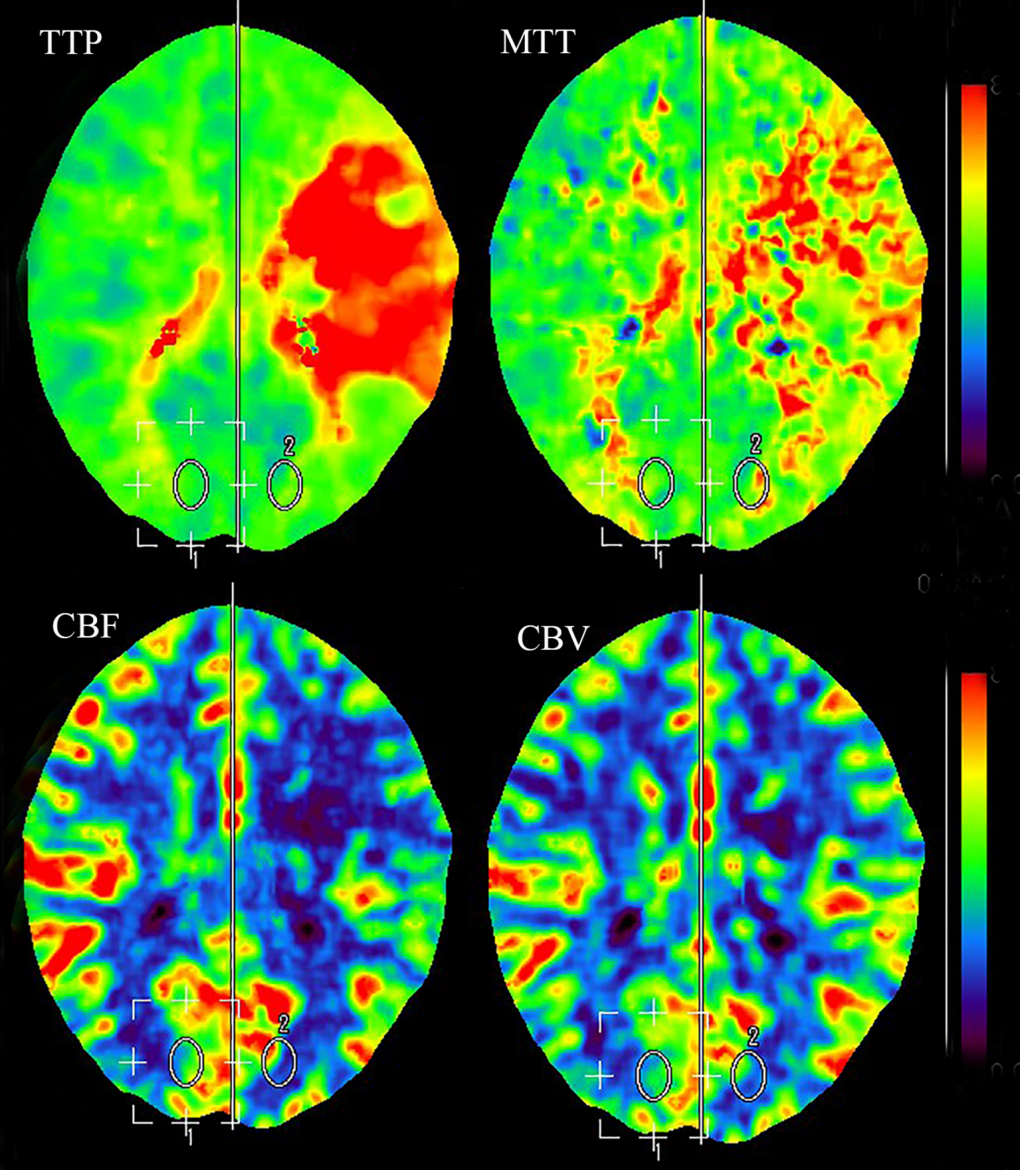
CT perfusion showing delayed TTP, increased MTT, reduced CBF, and preserved CBV in the left MCA region, suggesting a large salvageable ischaemic penumbra. CBF, cerebral blood flow; CBV, cerebral blood volume; CT, computed tomography; MCA, middle cerebral artery; MTT, mean transit time; TTP, time to peak.

**Figure 4:**
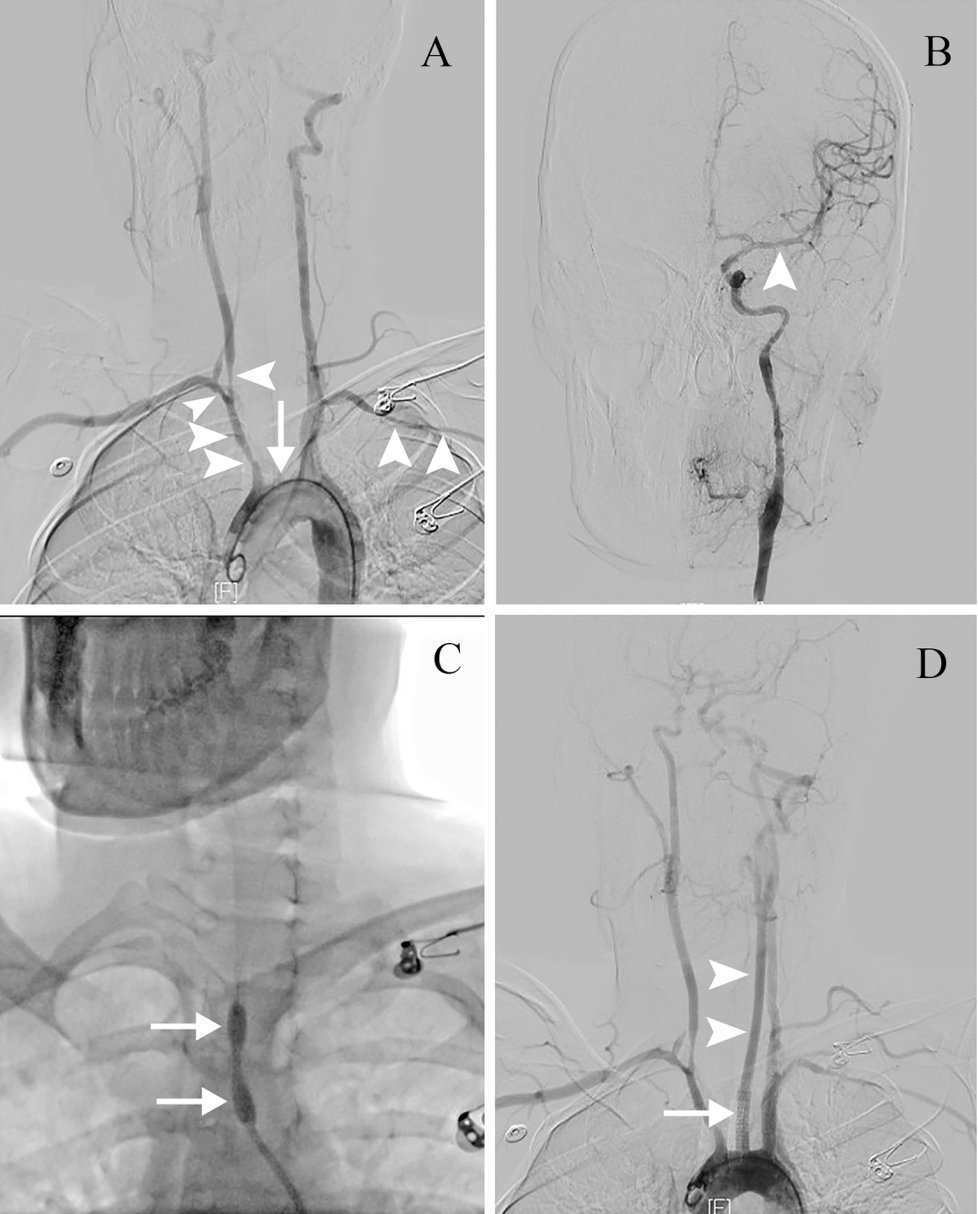
The endovascular therapy procedure. **(A)** DSA showing multiple stenoses (arrowheads) in the brachiocephalic trunk, the beginning of the right CCA and the bilateral subclavian arteries, with occlusion of the left CCA and its branches (arrow). **(B)** After mechanical thrombectomy, revascularisation of the left MCA is seen with TIMI flow grade 3 (arrowhead). **(C)** Percutaneous transluminal balloon angioplasty of the left CCA (arrows). **(D)** Stent implantation of the proximal left CCA (arrow). Revascularisation of the left CCA is seen (arrowheads). CCA, common carotid artery; DSA, digital subtraction angiography; MCA, middle cerebral artery; TIMI, Thrombolysis in Myocardial Infarction.

**Figure 5:**
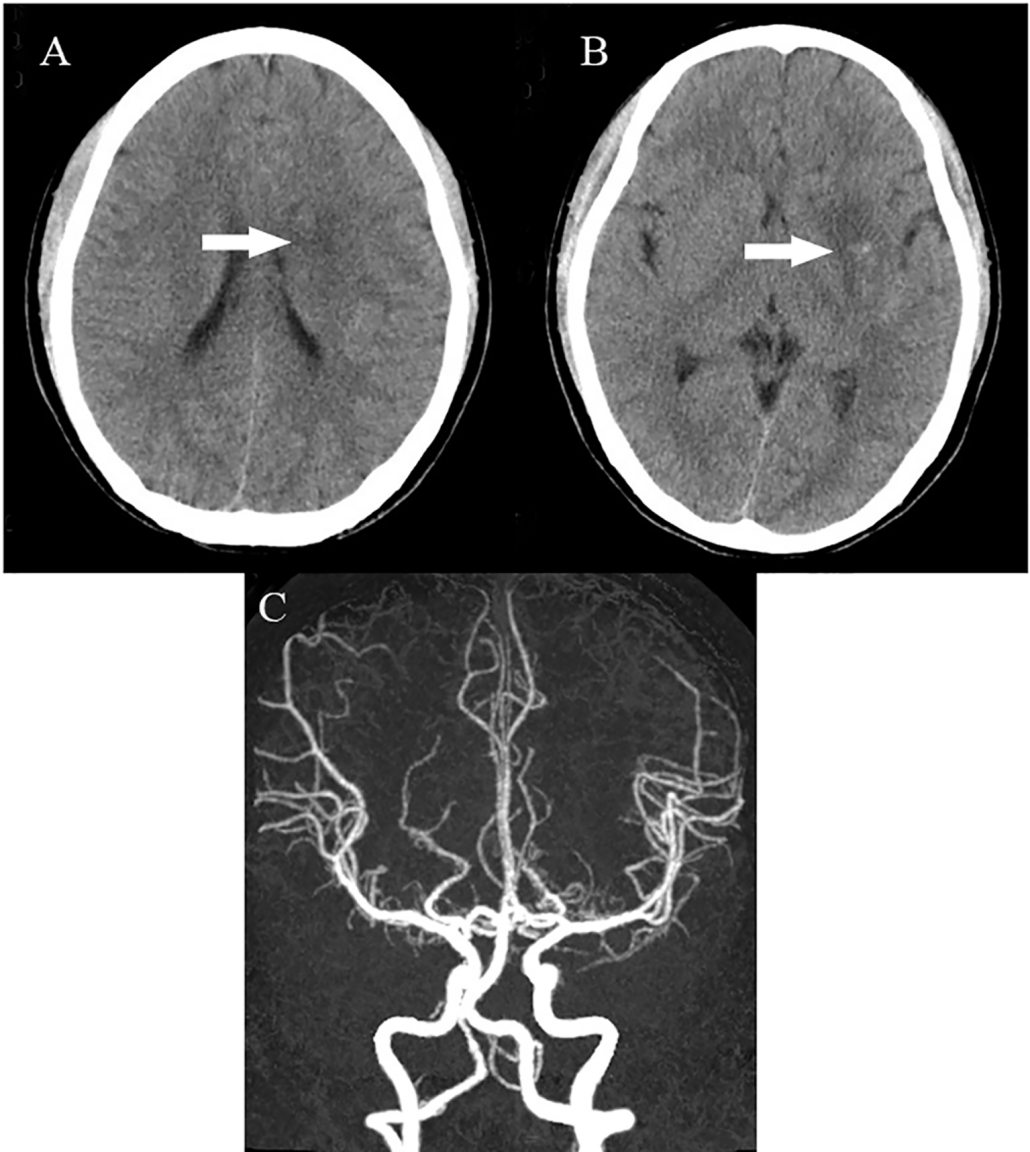
Head CT showing low-density lesions in the right basal ganglia **(A, B)** and mild high density inside the lesion **(B)** at 24 hours after endovascular therapy, suggesting a small core infarct area and contrast medium residue. Head CTA showing revascularisation of the left MCA **(C)**. CT, computed tomography; CTA, computed tomography angiography; MCA, middle cerebral artery.

Less than 3 hours had elapsed since the onset of symptoms, and the patient had no contraindications to recombinant tissue plasminogen activator (rt-PA) therapy. Therefore, rt-PA was initiated (4.05 mg bolus of alteplase followed by 36.45 mg over 1 hour at a total dose of 40.5 mg [0.9 mg/kg]), and she was transferred to our stroke unit. The patient’s symptoms resolved partially with an NIHSS score of 4 at 3 hours after onset (**Figure 1**). However, her neurological function deteriorated with an NIHSS score of 9 at 6 hours after onset. Computed tomography angiography (CTA) of the head showed asymmetry of the internal carotid arteries (left smaller than the right) and an abrupt occlusion of the M1 branch of the left MCA (**Figure 2B**). On CT perfusion, there was a delayed time to peak (TTP), increased mean transit time (MTT), reduced cerebral blood flow (CBF), and preserved cerebral blood volume (CBV) in the left MCA region, suggesting a large salvageable ischaemic penumbra (**Figure 3**). The pati was taken to the interventional operating room for emergency cerebral angiography 7 hours after the onset. Digital subtraction angiography (DSA) showed multiple stenoses in the brachiocephalic trunk, the beginning of the right common carotid artery (CCA), and the bilateral subclavian arteries, as well as occlusion of the left CCA and its branches (**Figure 4A**). The aorta was normal. Mechanical thrombectomy of the left MCA was performed immediately. Using the coaxial technique, an 8F guide catheter was placed into the end of the C1 segment of the left internal carotid artery (ICA), and a microwire/microcatheter (0.014 Traxcess, Headway-21) passed through the thrombus. The microcatheter angiography showed that the M2 segment of the left MCA was unobstructed to distal blood flow. A thrombectomy stent (Trevo retriever 4 mm × 20 mm) was implanted into the M1 segment. Following this, the microcatheter was withdrawn, the stent was released and removed after a static 5-min interval, and the thrombus (6 mm × 4 mm) was removed. Revascularisation of the left MCA was achieved with TICI flow grade 3 (**Figure 4B**). Percutaneous transluminal balloon angioplasty of the left CCA followed by stent implantation of the proximal left CCA was performed (**Figures 4C, D**). A protective umbrella (Filterwire EZ) was placed into the beginning of the left ICA, and a balloon dilatation stent (express LD, 6 mm × 28 mm) was placed along the protective umbrella at the opening of the left CCA to release the stent. The dilatation was performed 3 times. The blood flow of the left CCA, left MCA and left anterior cerebral artery was unobstructed, and the forward blood flow was TICI grade 3. There were no postoperative complications. Repeated head CT revealed low-density lesions in the right basal ganglia (**Figures 5A, B**) and a mild high density inside the lesion (**Figure 5B**) at 24 h after the endovascular therapy, suggesting a small core infarct area and contrast medium residue. Head CTA showed revascularisation of the left MCA (**Figure 5C**).

Since our patient had no risk factors for arteriosclerosis, she was further evaluated for the underlying aetiology for large vessel stenoses and occlusions. Blood tests showed an erythrocyte sedimentation rate of 16 mm/hr and a C-reactive protein (CRP) level of 66.20 mg/L. Rheumatological screening demonstrated seronegativity for rheumatoid factor, anti-cyclic citrullinated peptide, antinuclear antibodies, anti-neutrophil cytoplasmic antibodies, and anti-extractable nuclear antigens. Pulmonary arteries were normal on CT. Doppler vascular ultrasound revealed diffusely thickened arterial walls with an uneven low echo and uneven stenotic lumina at the beginning of the right CCA and the right subclavian artery. Limb and kidney vascular ultrasonography showed no abnormal findings. The echocardiogram and cerebrospinal fluid analyses were normal.

A diagnosis of TA was made based on the demographic features, differences in blood pressure, absent carotid pulse, elevated CRP, cerebrovascular event, and large vessel stenoses and occlusions. The patient was treated with intravenous methylprednisolone (1000 mg/day for 5 days, followed by 40 mg/day), aspirin (300 mg loading dose, then 100 mg/day), clopidogrel (300 mg loading dose, then 75 mg/day), and atorvastatin (40 mg/day). She was discharged home after 10 days with an NIHSS score of 1. Methylprednisolone (16 mg/day), azathioprine (100 mg/day), aspirin (100 mg/day), and atorvastatin (20 mg/day) were introduced as extended duration treatments (**Figure 1**). Her symptoms, including hemiplegia, facial nerve palsy, and aphasia, gradually improved. After 2 months post discharge, she recovered completely with no neurological sequelae. Her modified Rankin score and NIHSS score were both 0 at the 3-month follow-up. Blood flow was normal, and restenosis was not found on left carotid artery CT examination at the 1-year follow-up. Brain magnetic resonance imaging showed an old infarct lesion in the left basal ganglia and normal intracranial arteries.

## Discussion

This is a rare case of AIS as the first manifestation of TA. After emergency bridging therapy, the patient’s neurological deficit fully recovered without post-treatment complications. Bridging therapy may be an effective and safe treatment for AIS in patients with TA.

TA is an inflammatory vascular disorder that involves large vessels, predominantly the aorta and its main branches (1). The majority (80–90%) of cases occur in women, with an age of onset that is usually between 10 and 40 years (2). The entire course of TA involves two overlapping phases (6). Phase I is a systemic and asymptomatic phase. Phase II refers to vascular inflammation and/or sequelae such as stenosis. Involvement of the carotid and vertebral arteries causes decreased CBF, resulting in dizziness, vertigo, syncope, ataxia, headaches, seizures, and strokes. Previous studies found that 10-20% of people with TA have stroke events (3, 4). However, reports of stroke as the first manifestation of TA are rare, with few known case reports (7–9). In a retrospective study of 274 patients with TA, only 7 patients (2.6%) experienced stroke as an initial manifestation (10). Cerebrovascular events can be a significant cause of mortality in patients with TA (11). The underlying mechanisms of ischaemic stroke in patients with TA are diverse: artery-to-artery embolism from the aortic arch and its major branches, carotid aneurysm, carotid stump embolism, a hypercoagulable state, embolism due to aortic regurgitation, and secondary hypoperfusion (3, 12, 13). In this report, we present a rare case of TA with AIS as the first manifestation.

The mainstay of therapy for TA includes glucocorticoids, immunosuppressive agents, and biological agents (14). However, endovascular interventions or other surgical procedures may be required in phase II cases of irreversible arterial stenosis and occlusion, leading to large aneurysms or severe ischaemia. The approach of choice for vascular procedures in patients with TA is dependent on the anatomical location of the vascular lesion, timing of procedure (elective vs emergency), and other factors. The principal indications for surgery are as follows: uncontrolled hypertension related to renal artery stenosis, coarctation of the aorta, aortic regurgitation, ischaemic heart disease, a supra-aortic disease with symptomatic cerebrovascular disease, mesenteric ischaemia, severe limb extremity claudication, and aneurysm repair (15). Performing invasive procedures in patients with active conditions is linked to an increased risk of complications and lower patency rates (16, 17). Current studies suggest that the pathology of TA is characterised by the involvement of all arterial layers with a variable inflammatory infiltrate (18, 19). Acute, chronic, and granulomatous inflammation is observed mainly in the media and adventitia, while hyperplasia and neovascularisation are found in the intimal layer (1). In the active inflammation condition, the inflammation commences from the adventitia and progresses to the intima and leads to stenosis, occlusion, dilatation, or aneurysm formation (20). Previous studies have reported that stenting causes chronic static stresses and strains, leading to inflammation and cellular proliferation, causing arterial injury (21, 22). Arterial injury after percutaneous transluminal balloon angioplasty can be considered as not only neointima formation, but also as arterial remodelling (23). The actively inflamed arterial wall responds poorly to endovascular intervention, causing early restenosis or development of complications. Interventions should preferably be performed only after stable control of inflammation, except for emergency indications such as severe ischaemia and neurological complications such as aneurysm, dissection, or stroke (14). Endovascular intervention may still be a safe and effective method, although it involves technical problems and may cause complications (24). The majority of complications with endovascular treatment were technical failures followed by dissection of the vessels (25). Other complications included cerebral hyperperfusion syndrome, stent graft thrombosis, pseudoaneurysm, puncture site bleeding, stroke, restenosis, and renal failure. A 10-year retrospective study reported a complication rate of 8.2% (7/85) in an endovascular intervention group (24). Our patient with TA had obvious indications for emergency bridging therapy because her severe neurological deficits were related to AIS.

There are no randomised clinical trials regarding bridging therapy for AIS in TA. However, a few cases of emergency intravenous thrombolysis and/or mechanical thrombectomy have been reported (**Table 1**). Hedna et al. reported a woman presenting with an AIS treated with intravenous thrombolysis and subsequent endovascular intervention with a favourable outcome, followed by treatment with immunosuppressive agents after a diagnosis of TA (26). Furthermore, Komatina et al. reported a patient with TA, who subsequently developed AIS successfully treated with intravenous thrombolysis (27). Recently, Shan et al. reported that hybrid vascular reconstruction was a novel approach for achieving emergency revascularisation in an AIS patient with active TA (28). Additionally, Field et al. (29) and Davari et al. (8) illustrated a case of AIS in a patient with TA receiving intravenous thrombolysis. Although the symptoms of these patients recurred, mechanical thrombectomy was not performed due to concerns related to the friability of the vessels. Here, we presented a case of a 23-year-old woman with TA who presented with an AIS treated with bridging therapy. After careful consideration, percutaneous transluminal balloon angioplasty followed by stent implantation of the left CCA was performed because of the high thrombus burden and complete occlusion of the left CCA.

**Table 1:** Emergency mechanical thrombectomy and/or intravenous thrombolysis for AIS as a manifestation of TA.

Case	Age, years	Sex	Neurological symptoms	Intracranial offending arteries	Treatment for AIS	Treatment for TA	Follow-up
1 (26)	18	Female	Weakness, hemi-neglect	Right MCA	Bridging therapy	Glucocorticoids, biological agents	Good (17 months)
2 (27)	61	Female	Aphasia, weakness	Left MCA	rt-PA	Glucocorticoids	Good (6 months)
3 (28)	25	Female	Hemiparesis, speech disturbance	Left MCA	Hybrid vascular reconstruction	Glucocorticoids, biological agents	Good (6 months)
4 (29)	51	Female	Weakness, confusion, aphasia	Left MCA	rt-PA	Glucocorticoids, immunosuppressive agents	Stroke recurrence (2 days)
5 (8)	14	Female	Weakness, facial drooping, confusion	Left MCA	rt-PA	Glucocorticoids, immunosuppressive agents	Brain hernia (1 day)
6 (our case)	23	Female	Weakness, facial palsy, aphasia	Left MCA	Bridging therapy	Glucocorticoids, immunosuppressive agents	Good (12 months)

AIS, acute ischaemic stroke; MCA, middle cerebral artery; rt-PA, recombinant tissue plasminogen activator; TA, Takayasu arteritis.

The patient’s neurological function completely recovered. CTA and DSA showed that the left MCA was smooth, suggesting that the ischaemic vessel wall was not directly affected by the vasculitic process. Thrombosis of the left MCA was the reason for major AIS in our patient, probably due to carotid stump embolism and secondary hypoperfusion. After the diagnosis of TA was confirmed, glucocorticoids and immunosuppressive agents were administered immediately. The patient did not show any signs of restenosis during the short-term follow-up. However, restenosis and reintervention rates remain high during long-term follow-up, although long-term survival rates following endovascular intervention are promising (30).

In conclusion, TA must be recognised as a potential cause of stroke in young adults. Bridging therapy should be considered as a treatment option for AIS in patients with TA. Further study is needed in this regard.

## Data Availability Statement

All datasets generated for this study are included in the article.

## Ethics Statement

This study was approved by the Medical Ethics Committee of the Third Affiliated Hospital of Sun Yat-sen University. The participants' next of kin provided their written informed consent to participate in this study. Written informed consent was also obtained for the publication of any potentially identifiable images or data included in this article.

## Author Contributions

YD, YZ, and BZ designed the research. BJ and PL performed the experiments and analysed the data. PL, KS, and YL wrote the main manuscript text and prepared the figures. AM and BZ edited and revised the manuscript. All authors contributed to the article and approved the submitted version.

## Funding

This study was supported by the Guangdong Basic and Applied Basic Research Foundation (2020A1515010056), the Third Affiliated Hospital of Sun Yat-Sen University, Clinical Research Program (QHJH201907), and the Doctorial Starting Fund of Guangzhou Women and Children’s Medical Centre (2018-293).

## Conflict of Interest

The authors declare that the research was conducted in the absence of any commercial or financial relationships that could be construed as a potential conflict of interest.
